# Association between Low-density lipoprotein cholesterol and occipital periventricular hyperintensities in a group of Chinese patients: an observational study

**DOI:** 10.1186/s12944-017-0436-3

**Published:** 2017-02-27

**Authors:** Dazhi Duan, Lin Shen, Chun Cui, Tongsheng Shu, Jian Zheng

**Affiliations:** 1 Department of Neurology, Xinqiao Hospital, Third Military Medical University, No. 183, Xinqiao Street, Chongqing, 400037 China; 2 Department of Radiology, Xinqiao Hospital, Third Military Medical University, No. 183, Xinqiao Street, Chongqing, 400037 China

**Keywords:** MRI, White matter hyperintensity, Occipital periventricular hyperintensities, Low-density lipoprotein cholesterol, Cardiovascular risk factor

## Abstract

**Background:**

While occipital periventricular hyperintensities (OPVHs) are among the most common mild white matter hyperintensities, the clinical factors associated with OPVHs remain unclear. In this study, we investigated the role of clinical factors in development of pure OPVHs.

**Methods:**

This study included 97 patients with OPVHs and 73 healthy controls. Univariate analysis of clinical factors in OPVH patients and controls was followed by binomial logistic regression analysis to identify clinical factors significantly associated with OPVHs.

**Result:**

Univariate analysis indicated that age, total cholesterol (TC), low-density lipoprotein cholesterol (LDL-C) and apolipoprotein-B (Apo-B) levels differed significantly between the OPVH patients and controls (*p* < 0.05). Age and gender were correlated with OPVH scores (*p* < 0.05), while LDL-C, triglycerides, Apo-B and TC were anti-correlated with OPVHs scores (*p* < 0.05). Multivariate analysis indicated that LDL-C is negatively correlated with OPVHs (*p* < 0.05), and age is positively correlated with OPVHs (*p* < 0.001).

**Conclusion:**

In summary, LDL-C was negatively and age was positively associated with OPVHs among Chinese patients in a hospital.

## Background

White matter hyperintensities (WMHs), which reflect chronic vascular ischemic diseases, may only represent extreme cases of white matter injury [1]. Previous studies have investigated WMH progression and its clinical significance [2]. However, the optimal treatment regimen remains to be elucidated [3]. WMH can be categorized as periventricular hyperintensities (PVHs) or deep white matter hyperintensities (DWMHs) [4]. As the severity of the lesions increase, PVHs can extend into the deep white matter (DWM) and may subsequently develop into confluence DWMHs [5]. The precise differences between PVHs and DWMHs have not yet been fully explored, though some studies have suggested that their clinical and pathological features differ [6]. PVHs have also been suggested to be associated with brain atrophy, while DWMH is related to cerebrovascular events [7]. PVHs typically have caps around the occipital or frontal horns of the lateral ventricles and a pencil-thin lining or a smooth halo along the side of the lateral ventricles [8]. Thus, WMHs is often parcellated based on anatomy, such as occipital, parietal, frontal, and temporal lobes, as well as in fratentorial and basal ganglia regions, to assess its impact [9]. Such classifications can also reflect the different pathophysiological mechanisms of WMHs, and may be used to inform the design of clinical interventions. In addition, clarifying differences between PVHs and DWMHs may provide insights in the clinical significance of WMHs, and differences between pathological aging and normal aging.

Previous studies have reported that age and hypertension are common risk factors for WMHs [10, 11]. In contrast, the results of studies implicating other potential risk factors associated with WMH, such as diabetes mellitus, hyperlipidemia, smoking, high body mass index, vitamin B12 deficiency, and alcohol consumption, were inconsistent [10, 12–17]. Some studies have reported that hyperlipidemia has a protective effect on WMHs, however, these studies included mixed lesions which did not differentiate between early or mild lesions [18]. To our knowledge, the clinical factors contributing to WMHs at a single site have not yet been addressed. With reference to the aforementioned studies, we hypothesized that age is a risk factor while lipids and/or lipoproteins could have a protective effect on WMHs. In this case–control study, we investigated the role of clinical factors in development of pure OPVHs at a single site.

## Methods

### Subjects

Head magnetic resonance imaging (MRI) images were obtained in a total of 170 subjects from the department of neurology in Xinqiao Hospital, Third Military Medical University (age range: 55–85 years old). Images with Occipital periventricular hyperintensities (OPVHs) were identified according to the inclusion exclusion criteria. Their respective clinical data were then collected, and the patients were examined. The subjects were restricted from vitamin B12 intake for 3 months prior to scanning. Subjects with other diseases that can cause white matter lesions, such as toxic cerebral white matter lesions, multiple sclerosis, atrial fibrillation, progressive multifocal leukoencephalopathy and thyroid disease, were excluded from the study. In addition, we also excluded patients with cerebrovascular disease induced by heart and aorta embolism, atrial fibrillation, valvulopathy, myocardiopathy, endocarditis, left atrioventricular valve stenosis or ventricular aneurysm, vasculitis, familial high homocysteine, infectious diseases, anemia or malignant disease, intracranial tumors, systemic diseases, immune system diseases such as connective tissue or autoimmunity diseases, and those receiving treatments such as radiotherapy, drug chemotherapy and biological therapy.

Subjects were classified into two groups, OPVHs group (*n* = 97 with OPVHs ≥ 1 and without lesions in other locations) and control group (*n* = 73 with no WMHs) according to the Fazekas scale. No subjects had cerebral infarcts, defined as focal hyperintensities in T2 images. The gender (Gen), age, hypertension (Htn) and history of diabetes mellitus (DM), current smoking (CS) and alcohol use (CAU) were obtained with a patient-administered questionnaire. Total cholesterol (TC), high-density lipoprotein cholesterol (HDL-C), low-density lipoprotein cholesterol (LDL-C), triglycerides (TG), uric acid (UA), apolipoprotein-A1 (Apo-A1) and apolipoprotein-B (Apo-B) were also obtained. Note that this is a retrospective study, and the MRI images were obtained between 2012 October and 2015 October.

### MRI protocols

All MRI data were obtained using a 3.0 Tesla scanner (General Electric, Milwaukee, WI, USA) with a 12-channel head coil. FSE T2-weighted images and FLAIRT1-weighted images were acquired with TE/TR =112.2/3160 ms and TE/IT/TR =27.072/860/1696.68 ms, respectively. All MRI images were acquired with voxel size of 0.4688 × 0.4688 × 5 mm3, 20 sagittal slices and an in-plane resolution of 512 × 512. MRI images were then assessed visually by two neurologist using the Fazekas scale [19].

### Data analysis

Demographic and clinical variables were statistically analyzed using SPSS20 (IBMSPSS, Chicago, USA). Data are presented as mean and standard deviation. Chi-square test and student’s *t*-test were used to determine significant differences between frequencies of categories and continuous variables of the groups. In addition, the relationship between these factors and OPVH scores were analyzed using bivariate Pearson Correlation analysis. Subsequently, binomial logistic regression analysis and likelihood ratio test were performed to identify factors independently associated with OPVHs. To access and compare the sensitivity and specificity of using age and LDL-C as diagnostic factors, the receiver operating characteristic (ROC) curve was plotted using OPVHs as positive outcome, and age and LDL-C as diagnostic factors. Subsequently, the Youden Indices were calculated and the maximum Youden Index was used as cut-off value in the ROC curve [20–22]. The cut-off values for age and LDL-C were 57 years and 2.645 mmol/L, respectively. Using these cut-off values, the sensitivity and specificity were obtained. Finally, the area under ROC curve was quantified and statistically analyzed using SPSS. Results were considered significant when *P* < 0.05.

## Results

### T2-weighted MRI changes in OPVH patients

**Fig. 1:**
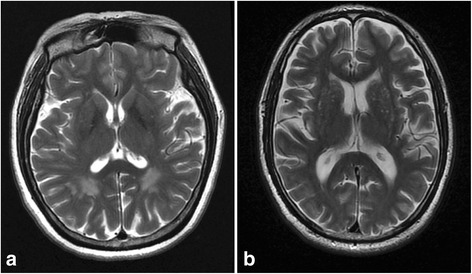
Typical T2-weighted MRI images a control and a OPVHs patient. Two typical T2-weighted MRI images of a control (**a**) and a OPVHs patient (**b**). High-signal intensities are observed on the T2-weighted image of the OPVHs patient. In addition, no cerebral infarcts were observed on the T2-weighted images in any subjects

Figure 1 shows two typical T2-weighted MRI images of a control (Fig. 1a) and a OPVHs patient (Fig. 1b). High-signal intensities were observed on the T2-weighted images of OPVHs patients. In addition, no cerebral infarcts were observed on the T2-weighted images in all subjects.

### Clinical variables and OPVHs using univariate analysis

Table 1 summarizes the differences between the OPVH patients and controls. The OPVH patients were significantly older (*p* < 0.001), with lower TC (*p* <0.05), LDL-C (*p* < 0.001) and Apo-B (*p* < 0.05) levels. Table 2 summarizes the correlations between OPVHs and potential risk factors. Spearman correlation analysis indicated that age (*p* < 0.001) and gender (*p* < 0.05) were correlated with OPVHs, while LDL-C (*p* < 0.001), TG (*p* < 0.05), Apo-B (*p* < 0.001) and TC (*p* < 0.05) were anti-correlated with OPVHs.

**Table 1:** Clinical factors differing between OPVHs and CN

Group	OPVHs	CN	*χ*2/t	*p*
*n*	97.00	73.00	/	/
OPVH Scores	2.72 (0.54)	0.00 (0.00)	/	/
Gen (*n*,% male)^1^	61(62.89)	35(47.95)	3.78	0.05
Age (years)^2^	71.79(8.29)	68.9(7.91)	−4.87	<0.001^*^
CS (*n*,%)^1^	24(24.74)	21(28.77)	0.35	0.56
CAU (*n*,%)^1^	21(21.65)	14(19.18)	0.16	0.69
Htn (*n*,%)^1^	47(48.45)	26(35.62)	2.80	0.09
DM (*n*,%)^1^	18(18.56)	8(10.96)	1.86	0.17
TC^2^	4.61(1.07)	4.81(0.96)	2.05	0.04^*^
TG^2^	1.44(0.76)	1.66(1.17)	1.48	0.14
LDL_C^2^	2.51(0.63)	2.83(0.68)	3.20	<0.001^*^
HDL_C^2^	1.34(0.32)	1.29(0.30)	−0.95	0.34
UA^2^	289(75.30)	277.75(67.33)	−1.01	0.31
Apo-A1^2^	1.30(0.31)	1.25(0.33)	−1.01	0.31
Apo-B^2^	0.84(0.24)	0.91(0.23)	2.24	0.03^*^

Continuous variables are presented as mean (standard deviation) and compared using Student’s *t*-test, while categorical variables are presented as percent and compared between groups using the chi-square test,. 1, 2 * denotes Chi-square test, Student’s *t*-test and *p* < 0.05, respectively

**Table 2:** Correlation of clinical factors and OPVHs

Risk factors	R	*P*
Age	0.36	<0.001^*^
Gen	0.18	0.02^*^
Htn	0.12	0.12
DM	0.07	0.34
CAU	0.05	0.51
CS	−0.04	0.59
UA	0.08	0.30
LDL-C	−0.25	<0.001^*^
HDL-C	0.07	0.35
TG	−0.17	0.03^*^
Apo-A1	0.07	0.39
Apo-B	−0.24	0.00^*^
TC	−0.17	0.03^*^

Statistical correlations were determined by two-tailed bivariate Pearson correlation coefficient test with all risk factors as independent variables and the OPVH scores as dependent variables. * denotes *p* < 0.05

### Clinical variables and OPVHs using multivariate analysis

**Fig. 2:**
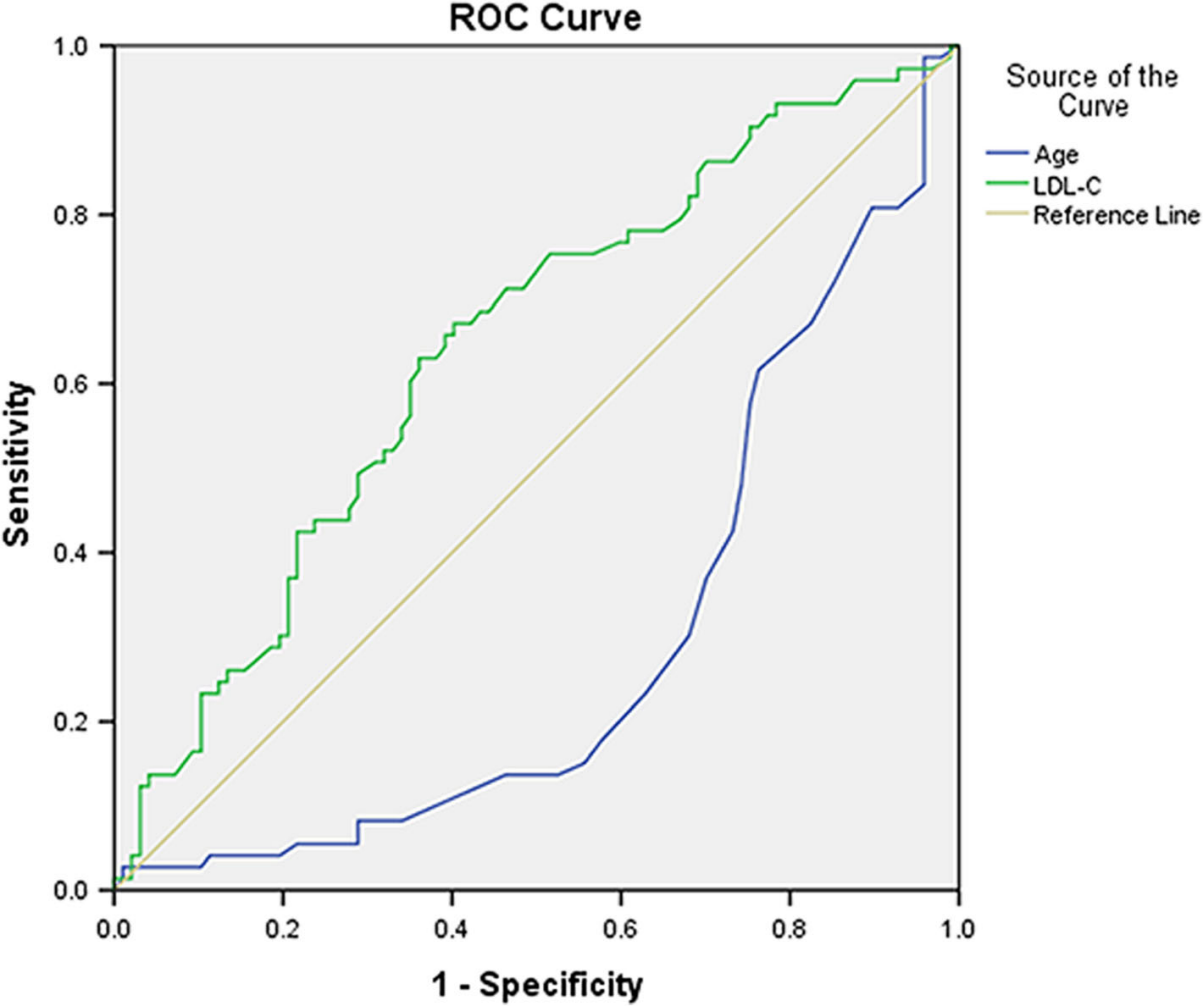
Receiver operating characteristic curve using age and LDL-C as diagnostic factors for OPVHs. ROC were obtained using OPVHs as positive outcome and age and LDL-C as diagnostic factors. It should be noted that the diagnostic strength of LDL-C, calculated using on the area under the curve, was greater than that of age for OPVHs

Binomial logistic regression analysis indicated that age (*p* < 0.001) was positively correlated with OPVHs, while LDL-C (*p* < 0.05) was negatively correlated with OPVHs (Table 3). For ROC analysis, the sensitivity and specificity of age as a diagnostic factor were 0.986 and 0.041, respectively. On the other hand, the sensitivity and specificity of LDL-C as a diagnostic factor were 0.630 and 0.639, respectively. The ROC indicated that age (*p* < 0.001) and LDL-C (*p* < 0.01) differed significantly between OPVH patients and healthy controls (Table 4). These results suggest that OPVHs and age are positively associated, while OPVHs and LDL-C are negatively associated, respectively. It should be noted that LDL-C was a better predictor of OPVHs than age, based on the area under the curve (Fig. 2).

**Table 3:** Binomial logistic regression analysis of Independent factors for OPVHs

Risk factors	B	*p*-value	OR	95% C. I. of OR	
				Lower Bound	Up Bound
Age	0.09	<0.001^*^	1.09	1.04	1.14
Gen	0.53	0.16	1.70	0.82	3.52
TC	0.43	0.26	1.53	0.73	3.20
TG	−0.18	0.33	0.84	0.59	1.20
LDL-C	−0.99	0.03^*^	0.37	0.16	0.89
Apo-B	−0.17	0.86	0.84	0.13	5.55

*P*-values were obtained using OPVHs as positive outcome and Age, Gen, TC, TG, LDL-C, Apo-B as independent variables by binomial Logistic regression analysis. * denotes *p* < 0.05

**Table 4:** Diagnostic strength of independent factors for OPVHs

Variable	Area	*p*-value	95% C. I. of Area	
			Lower Bound	Upper Bound
Age	0.290	<0.001^*^	0.211	0.368
LDL-C	0.642	0.002^*^	0.558	0.726

*P*-values and the area under the curve were obtained using OPVHs as positive outcome with age and LDL-C as diagnostic factor. * denotes *p* < 0.05

## Discussion

White matter hyperintensities (WMHs) are thought to reflect chronic cerebral small vessel diseases, and can be easily detected using MRI. In previous studies, age and hypertension were reported to be the most common risk factors for WMHs [10, 11], while the role of other potential risk factors, such as diabetes mellitus, hyperlipidemia, smoking, high body mass index, B12 deficiency, and alcohol were reported inconsistently [10, 12–17]. Our results demonstrate that age was strongly correlated with the occurrence of OPVHs, which is consistent with previous studies. In addition, LDL-C was anti-correlated with OPVHs. These results indicate that age was positively correlated to OPVHs, while LDL-C was negatively correlated with OPVHs.

Receiver operating characteristic curve (ROC), also known as susceptibility curve, sensitivity curve, or diagnostic curve, can reflect both sensitivity and specificity. The ROC curve is based on the probability of true positive rate versus false positive rate. Note that the closer the ROC curve is to the upper left, the higher the accuracy of the test. In other words, the point of the ROC curve closest to the upper left corner is the best threshold to be used with a minimal number of false positives and false negatives. The advantages of ROC curve are as follows. First, it can easily identify the diagnostic ability using an optimal diagnostic threshold. Second, ROC curve can differentiate two or more different diagnostic tests by plotting the ROC curves of each trial or diagnostic test in the same plot to visually identify the advantages and disadvantages of these diagnoses. Third, it is possible to compare the area under the ROC curve (AUC) for each method, which the larger that AUC the better, to identify the best diagnostic method [21, 23]. In order to study the sensitivity and specificity of LDL-C in diagnosing OPVHs, we performed ROC curve analysis. Our results indicate that LDL-C was a better predictor of OPVHs than age, since the sum of sensitivity and specificity of LDL-C is higher than that of age. Despite the advantages of ROC curve analysis, it should be noted that high AUC values may actually have poor overall accuracy in some cases.

LDL-C is widely recognized as a risk factor for stroke [24, 25], and lipid-lowering therapies have demonstrated benefits in stroke prevention and prognosis [26, 27]. Statin treatment may protect the vessels of the brain and increase survival rate. However, previous studies have suggested that statin treatment may deteriorate WMHs [28]. We observed that higher LDL-C levels were associated with lower severity of OPVHs, which is consistent with previous findings [29, 30]. Our results also highlighted other less influential factors, with LDL-C being a more influential factor than age. Although the mechanisms underlying the anti-correlation between LDL-C and OPVHs are not fully understood, we speculate that cholesterol may play an important role in neuron repair and remodeling in the central nervous system [31].

Previous studies of the effect of pravastatin on the progression of ischemic brain lesions were evaluated using MRI longitudinally [32]. After treatment, no difference in lesion progression was observed between the pravastatin-treated and placebo groups. In another study involving participants with long-standing type 2 diabetes at a high risk for cardiovascular events, decreased LDL-C levels did not produce significant cognitive changes at 40 months of follow-up [33]. These studies suggest that lowering cholesterol did not benefit the white matter or cognitive functions. However, our results suggest that decreasing LDL-C could deteriorate WMHs. It should be noted that our study focused on mild OPVHs, while previous studies included more severe OPVHs. This might explain the discrepancy between our results and those of previous studies, since early mild lesions should have less influence factors. Our study also provides insights that may inform development of simple preventive measures.

WMHs are largely divided into PVHs and deep DWMHs [4], but the precise difference between PVHs and DWMHs is yet to be fully elucidated, with both types strongly associated with age [34]. Previous studies have suggested that the clinical and pathological features of these two types of WMHs are diverse [6]. PVHs may be more strongly affected by additional factors related to aging, such as hypotension, hypoperfusion, and atrophy [35–37], whereas DWMHs may be associated with cerebrovascular events [7]. Previous studies indicate that a reduction in total cerebral blood flow is associated with increased PVH volume but not DWMH volume [38]. It is also known that cholesterol can play a fundamental role in the development of the central nervous system and in the creation and maintenance of new synapses, which may be related to OPVHs [31, 39, 40]. Hypoperfusion may reduce delivery of nutrients to the white matter in the brain [41]. The periventricular white matter extends 3 to 13 mm around the periventricle [42], the blood supply of this area is provided by choroidal arteries or the terminal branch of lenticulostriate arteries of subependymal artery [43], anastomose of the vessels is sparse or missing, and the area is prone to ischemia during cerebral ischemia or hypoperfusion [41]. Surprisingly, accumulating evidence suggests that it is low rather than high levels of circulating cholesterol that may contribute to the risk of primary intracerebral hemorrhage [44] and to a higher mortality rate in these patients [45]. Furthermore, it has been shown that higher cholesterol levels are associated with less severe WMHs [46–48]. This could explain the role of elevated cholesterol levels and a better response to an acute injury such as stroke, as well as better responses to chronic cerebral injury (such as the processes involved in WMH development). Our results, along with these previous studies, support the hypothesis that hypercholesterolemia may play a protective role in cerebral small-vessel disease [49], whereas hypocholesterolemia appears to impair endothelial reparative processes [50]. Both lower LDL-C and severity of WMH have a significant heritability component [51, 52]. Combined with previous research, our results show that higher cholesterol was associated with reduced OPVHs, and relatively higher cholesterol might be associated with better health status in late life [53]. It should be noted that abnormally high cholesterol levels are not associated with better health. Our study provides a basis for the selection of populations that require intervention and intervention methods, in particular the widespread use of statins should be carefully reviewed. Our results also offer a model for the investigation of WMH pathogenesis in future animal studies.

The limitations of this study relate first to its retrospective design, and second to the small sample size. A specifically designed, randomized, controlled prospective population-based study may ultimately clarify the role of LDL-C in OPVHs, and is warranted in future studies. Our ROC analysis revealed that using age as the diagnostic factor of OPVHs was mathematically correct, however, it may have few implications as the results showed a low specificity. Future studies may investigate other parameters as diagnostic factors of OPVHs. Also, our findings are only from a Chinese hospital and do not rule out the possibility of selective bias or sampling error. In addition, future studies may encompass the mechanisms on how LDL-C affects OPVHs using animal models. Although body mass index and VB12 are associated with OPVHs, how these factors exactly affect OPVHs remains to be elucidated.

## Conclusions

LDL-C was negatively, and age was positively associated with OPVHs among Chinese patients in a hospital.

## Abbreviations

Apo-A1: Apolipoprotein-A
Apo-B: Apolipoprotein-B
C. I: Confidence interval
CAU: Current alcohol use
CN: Controls
CS: Current smoking
DM: Diabetes mellitus
Gen: Gender
HDL-C: High density lipoprotein cholesterol
Htn: Hypertension
LDL-C: Low density lipoprotein cholesterol
OPVHs: Occipital periventricular hyperintensities
ROC: Receiver operating characteristic
TC: Total cholesterol
TG: Triglyceride
UA: Uric acid
WMHs: White matter hyper intensities
